# Clinical experience and satisfaction in patients with advanced breast cancer participating in the abemaciclib patient support program in Spain: a prospective observational study

**DOI:** 10.1007/s12094-026-04267-y

**Published:** 2026-03-12

**Authors:** Isabel Blancas, Miriam González de la Peña, María Fernández Abad, Silvia Antolín Novoa, Encarna Adrover Cebrián, Rodrigo Sánchez Bayona, Esther Zamora Adelantado, Raquel Andrés Conejero, Sonia del Barco Berrón, Manuel Atienza, Alberto Molero, Silvia Díaz-Cerezo, Clara Pérez-Rambla, F. J. Pérez-Sádaba, Luis Manso

**Affiliations:** 1 Hospital Universitario Clínico San Cecilio, Granada, Spain; 2 Department of Medicine, Granada University, Granada, Spain; 3 Instituto de Investigación Biosanitaria Ibs.Granada, Granada, Spain; 4 Hospital Universitario de Jerez de la Frontera, Jerez de la Frontera, Cádiz, Spain; 5 Hospital Universitario Ramón y Cajal, Madrid, Spain; 6 INIBIC, Hospital Universitario de A Coruña, A Coruña, Spain; 7 Complejo Hospitalario Universitario De Albacete, Albacete, Spain; 8 Department of Medical Oncology, Hospital Universitario 12 de Octubre, Madrid, Spain; 9 Instituto de Investigación Sanitaria Hospital 12 de Octubre (imas12), Madrid, Spain; 10 Hospital Universitario Vall d’Hebron, Vall d’Hebron Institute of Oncology (VHIO), Barcelona, Spain; 11 Hospital Clínico Universitario Lozano Blesa, Zaragoza, Spain; 12 Instituto Catalán de Oncología, HU Doctor Josep Trueta, Girona, Spain; 13 Eli Lilly and Company, Indianapolis, USA; 14 Outcomes’10, a Product Life Group Company, Castellón de la Plana, Castellón Spain

**Keywords:** Patient support programs, Abemaciclib, Metastatic breast cancer, Diarrhea, Real world evidence

## Abstract

**Purpose:**

To evaluate the impact of a patient support program (PSP) on the management of abemaciclib-related diarrhea in patients with hormone receptor-positive, human epidermal growth factor receptor 2–negative (HR+/HER2−) metastatic breast cancer (MBC) and its influence on adherence and patient-reported outcomes in routine clinical practice.

**Methods:**

This is a multicenter, prospective, observational Spanish study in patients with locally advanced or MBC receiving abemaciclib and enrolled in the PSP, assessed over 6 months. The primary endpoint was the proportion of patients reducing or discontinuing abemaciclib due to diarrhea. The secondary endpoints included diarrhea-related temporary interruptions, diarrhea management, adherence, HRQoL, and satisfaction with the PSP. Descriptive statistics were applied and treatment modification endpoints were analyzed using Kaplan–Meier.

**Results:**

The study included 39 patients (median age: 58 years), with a median time since diagnosis of MBC of 2 months. Diarrhea occurred in 89.7% of patients, with grade 3 events in 7.7% and no grade ≥ 4 events. Nine patients (23.1%) experienced treatment modifications due to diarrhea; however, no permanent treatment discontinuations were reported. Loperamide (over 75% of patients) and dietary modifications were the most used self-care strategies. At week 24, results from the ad hoc questionnaires showed that over 70% of the patients reported high satisfaction with all PSP aspects, and 80% were classified as treatment adherent.

**Conclusions:**

Episodes of diarrhea were mostly graded 1–2 and no patients discontinued abemaciclib due to diarrhea. Patients reported high satisfaction with abemaciclib PSP, good adherence, and favorable quality of life, supporting the use of PSP in clinical practice.

**Supplementary Information:**

The online version contains supplementary material available at 10.1007/s12094-026-04267-y.

## Introduction

Breast cancer (BC) is the most prevalent cancer among women globally, representing approximately 30% of female cancer diagnoses [1], with over 2 million new cases annually [2]. Approximately 70% of the patients have hormone receptor-positive (HR+) and human epidermal growth factor receptor-negative (HER2−) BC [3], a subtype associated with better prognosis and favorable initial responses to endocrine therapy (ET) [4]. Despite this, 25–30% eventually develop metastatic disease (MBC) [5].

HR+/HER2− MBC remains incurable and ET is the standard treatment, although resistance is common [1, 6]. To improve outcomes in this setting, oral cyclin-dependent kinase 4 and 6 (CDK4/6) inhibitors like abemaciclib have become widely used [6–8]. In MONARCH-2 and -3, abemaciclib plus ET significantly improved progression-free survival but was frequently associated with diarrhea (over 80% of patients) and caused treatment discontinuation in 2–3% [9, 10].

Diarrhea and other adverse events (AEs) can compromise adherence, delay treatment, and lead to dose reductions or discontinuations [11]. The early identification and management of AEs—including education and prompt identification of low-grade toxicities, plus nutritional counseling and supportive care—are key to maintaining adherence and optimizing outcomes [11].

Patient support programs (PSPs) offer strategies to enable patients to manage their disease and associated adverse events. These programs often focus on individualized counseling and fostering effective communication between patients and healthcare professionals (HCPs), aiming to enhance treatment adherence, improve clinical outcomes, elevate the patient experience with treatment, and/or increase their quality of life [12].

In this context, in Spain, patients starting treatment with abemaciclib for MBC can voluntarily participate in a PSP sponsored by the marketing authorization holder. This program offers telephone education 24/7 by nurses in a call center through both outbound and inbound calls, providing guidance on self-care, nutrition, and management of side effects, particularly diarrhea. Nurses provide support, advice, and information, always referring the patient to the treating oncologist for any treatment-related decisions, such as dose changes or interruptions [13].

Despite the increasing availability of PSPs for oncological patients, there is a lack of published evidence on their quantifiable impact, particularly in the management of treatment-related AEs (12). This study aimed to assess the clinical impact of abemaciclib PSP on managing abemaciclib-related diarrhea. The findings may offer valuable insights into the potential benefits of these strategies for patients.

## Methods

### Study design and participant selection

A multicenter, prospective, observational study was conducted across nine Spanish hospitals in patients with locally advanced or MBC prescribed abemaciclib and registered in the PSP. PSP registration was voluntary, independent of the study and available to any patient treated with abemaciclib. The study inclusion criteria were: (i) age ≥ 18 years; (ii) diagnosis of locally advanced or MBC; (iii) prescription of abemaciclib; (iv) registration in the PSP before the first follow-up visit (15 days from treatment initiation, allowing a 7-day window); and (v) signed informed consent. Patients participating in clinical trials or other PSPs were excluded. The study had a 15-month recruitment period, from September 2022 to December 2023, and included a 6-month follow-up period, consisting of five visits that coincided with routine clinical visits at weeks 2, 4, 8, 12, and 24 (Supplementary Fig. 1).

The study was approved by the Hospital 12 de Octubre Ethics Committee (May 17, 2022) and local review boards and was registered with AEMPS (GESTO-REEC 0080-2022-OBS, No.: 22-0087). It complied with national regulations and the Declaration of Helsinki.

The primary objective was to evaluate the proportion of patients who reduced the dose or discontinued abemaciclib due to diarrhea during follow-up.

Additional objectives were to describe: (i) sociodemographic and clinical characteristics; (ii) temporary treatment interruptions due to diarrhea; (iii) management of diarrhea from both physician and patient perspectives; (iv) treatment adherence; (v) health-related quality of life (HRQoL); and (vi) persistence and satisfaction with the PSP.

### Data collection and study variables

Data were collected using a paper Case Report Form (CRF) designed for the study. The CRF consisted of two modules. The physician-reported module captured sociodemographic and clinical data (age, weight, height, body mass index (BMI), carcinoma type, metastasis status, time since diagnosis, follow-up duration, and comorbidities). It also documented frequency and type of stool (according to the Bristol scale), diarrhea episodes, and their severity (graded using common terminology criteria for adverse events (CTCAE)), initial abemaciclib dose, any dose reductions, interruptions or discontinuations, including their reasons. Additionally, concomitant antidiarrheal treatments, instructions provided, PSP enrollment and withdrawal dates, and treatment adherence, were also collected during routine visits.

The patient-reported module included HRQoL, ad hoc questionnaires on self-care and diarrhea management, and a PSP satisfaction questionnaire.

At visit 1 (week 2), the data were collected covering the period before the initiation of abemaciclib and the first 15 days of treatment. Clinical, treatment-related, adherence, and patient-reported data were collected during all the follow-up visits, when available.

### Patient-reported outcomes and related measures

#### Patients’ self-care and diarrhea management

This ad hoc questionnaire, completed by patients, consisted of two parts (Supplementary Table 1). The first included nine items on specific strategies used to manage diarrhea and identified whether these were recommended by an HCP, the PSP, or other sources. It also assessed the perceived effectiveness of each action. The second section included eight items assessing patients’ perceptions on their ability to manage diarrhea, rated on a 5-point Likert scale (from “Completely disagree” to “Completely agree”). At weeks 2 and 4, this questionnaire covered the period since the last study visit; at weeks 8, 12, and 24 it referred to the prior 2 weeks.

#### Treatment adherence (SMAQ)

Adherence to abemaciclib was assessed using the six-item Simplified Medication Adherence Questionnaire (SMAQ) [14], administered by physicians during study visits. The patients were classified as “non-adherent” if they responded with any non-adherent answer, missed more than two doses in the previous week or had not taken abemaciclib for more than two full days within the past three months, relative to the prescribed schedule; HCP-directed holds were excluded.

#### HRQoL assessment

HRQoL was assessed at all visits using the Functional Assessment of Chronic Illness Therapy-Diarrhea (FACIT-D) questionnaire, which evaluates four dimensions (physical, social/family, emotional, and functional) plus a diarrhea-specific subscale (DS) [15]. The responses were rated on a 0–4 Likert scale (“Not at all” to “Very Much”), with higher scores indicating a better HRQoL. The total FACIT-D score ranges from 0 to 152 and the DS from 0 to 44. The subscores were calculated when ≥ 50% of items were completed; 80% completion was required to calculate the total FACIT-D score.

#### Satisfaction with PSP

Patients completed an ad hoc questionnaire rating satisfaction on a 5-point Likert scale (from “Completely unsatisfied”, to “Completely satisfied”). The questionnaire also included the number of PSP consultations, and the topics discussed (nutritional or diarrhea management) (Supplementary Table 2).

### Sample size calculation and statistical analysis

Sample size was based on an expected 18.8% rate of dose reduction or discontinuation due to diarrhea, as reported in clinical trials [11]. Assuming an expected registration in the PSP of 10 patients per month over 6 months, and applying a 95% bilateral confidence interval with a precision error of 8%, the minimum required sample size was 37 patients.

The categorical variables were presented as absolute and relative frequencies, with the number of patients indicated for each category. Quantitative data were reported using mean and standard deviation (SD) or median and interquartile range (IQR). As the main objective of the study was descriptive, no inferential statistical comparisons or calculation of confidence intervals or *p* values were performed.

Time-to-event variables were defined as the number of days from treatment initiation to the first occurrence of each outcome: dose reduction, temporary interruption, or permanent discontinuation.

Survival analyses were conducted using the Kaplan–Meier method to estimate the probability of each event over time. Patients who did not experience the event by the end of follow-up were censored. All analyses were performed using STATA version 14.

## Results

### Sociodemographic and clinical characteristics

A total of 39 patients were enrolled, with a median age of 58 years (IQR 52.0–67.0) (Table 1). Most (94.9%) had metastatic breast cancer, with metastasis in visceral organs (*n* = 16), bone (*n* = 19), other locations (*n* = 13), and brain (*n* = 1), while 2 patients (5.1%) had locally advanced disease. At abemaciclib initiation, the median time since diagnosis of metastatic disease was 2.0 months. Patients registered in the PSP a mean of 7.7 days after the start of abemaciclib treatment, and most (94.9%) started at 150 mg twice daily (BID) dose. The median follow-up time was 5.6 months.

**Table 1:** Sociodemographic and clinical characteristics at initiation of abemaciclib treatment (*N* = 39)

Characteristics	Value
Age, median years (IQR)	58.00 (52.0–67.0)
BMI, mean (SD)	27.20 (4.95)
Normal (18.5 ≤ BMI < 25), *n* (%)	17 (43.59)
Overweight (25 ≤ BMI < 30), *n* (%)	12 (30.77)
With obesity (BMI ≥ 30), *n* (%)	10 (25.64)
Carcinoma type, *n* (%)	
Locally advanced BC	2 (5.13)
Metastatic BC*	37 (94.87)
Visceral	16 (41.03)
Bone	19 (48.72)
Brain	1 (2.56)
Other	13 (33.33)
Median time since diagnosis, months (IQR)	2.00 (1.0–26.0)
Median follow-up time, months (IQR)	5.59 (5.19–5.78)
Median time from start of abemaciclib to PSP registration, days (IQR)	7.00 (0.0–14.0)
Comorbidities, *n* (%)	
Yes	19 (48.72)
No	20 (51.28)
Abemaciclib starting dose, *n* (%)	
50 mg BID	1 (2.56)
100 mg BID	1 (2.56)
150 mg BID	37 (94.87)
Bowel movements (prior to start abemaciclib treatment). Number of daily bowel movements, *n* (%)**	
0	6 (18.18)
1	24 (72.73)
2	3 (9.09)
Type (according to the Bristol stool scale), *n* (%)***	
Type 1	1 (3.57)
Type 2	4 (14.29)
Type 3	9 (32.14)
Type 4	11 (39.29)
Type 5	1 (3.57)
Type 6	1 (3.57)
Type 7	1 (3.57)

*BC* breast cancer, *BMI* body mass index, *MBC* metastatic breast cancer, *PSP* patient support program, *SD* standard deviation

*Multiple responses were possible for metastatic sites. **n = 33, 6 missing values, ***n = 28, 11 missing values

#### Frequency and types of stools

Before starting abemaciclib, most participants reported regular bowel habits according to the Bristol scale: 72.7% had one daily bowel movement and 75% had normal stool types (3, 4, or 5) (Table 1).

Overall, 35 of the 39 participants (89.7%) experienced diarrhea during the study, including three patients (7.7%) with grade 3 events. No patients experienced grade 4–5 diarrhea. The median time to first diarrhea episode was 10 days (IQR 3–21) after the initiation of abemaciclib. All events were attributed to abemaciclib. The proportion of patients with diarrhea decreased from 62 to 50% from weeks 2 to 24, respectively (Table 2).

**Table 2:** Diarrhea events

	Number of patients					
Diarrhea	2 weeks (*n* = 39)	4 weeks (*n* = 38)	8 weeks (*n* = 38)	12 weeks (*n* = 38)	24 weeks (*n* = 36)	Total (*n* = 39)
Yes, n (%)	24 (61.54)	27 (71.05)	25 (65.79)	21 (55.26)	18 (50)	35 (89.74)
No, n (%)	15 (38.46)	11 (28.95)	13 (34.21)	17 (44.74)	18 (50)	4 (10.26)
MD	0	1	0	0	1	
*The reported n refers to patients continuing in the study. One patient discontinued at week 4, one at week 12, and four at week 24; therefore, n is adjusted accordingly for follow-up visits*						

Most severe grade per patient, *n* (%) (*n* = 39)		
CTCAE		
Grade 1	18 (46.15)	
Grade 2	14 (35.90)	
Grade 3	3 (7.69)	
Grade 4	0 (0)	
Grade 5	0 (0)	
No diarrhea	4	

*CTCAE* common terminology criteria for adverse events, *MD* missing data

#### Treatment modifications due to diarrhea

**Fig. 1:**
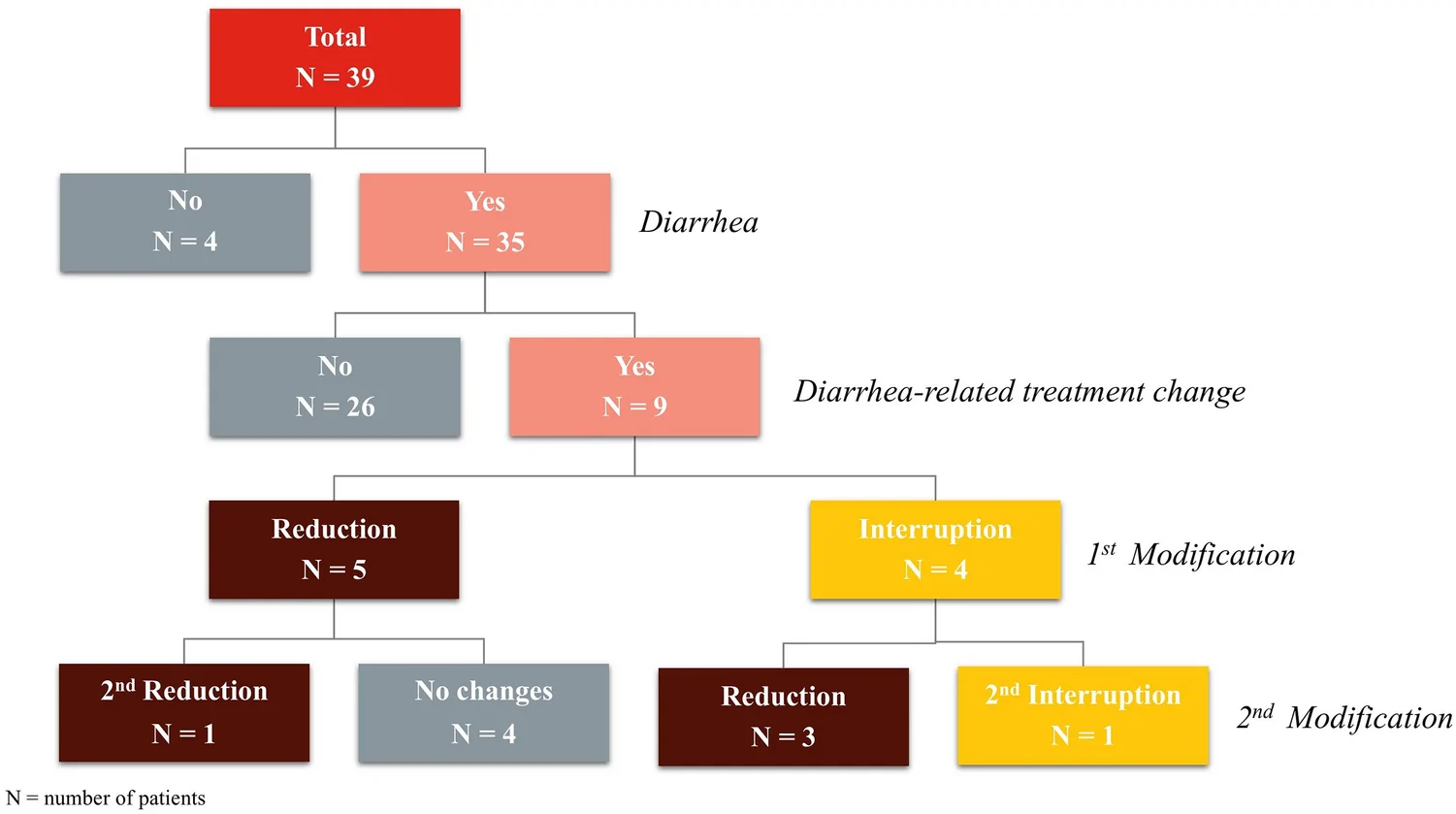
Diarrhea-related modifications to abemaciclib treatment

No patient discontinued abemaciclib due to diarrhea. Among the 35 patients with diarrhea, 9 (23.1%) modified their treatment, either reducing the dose and/or temporarily interrupting treatment on one or more occasions (Supplementary Fig. 2). These nine patients accounted for nine dose reductions and five temporary interruptions. Eight patients reduced the dose once (all from 150 mg BID to 100 mg BID), and one patient did so twice (eventually reducing to 50 mg BID). Three patients had temporary interruptions prior to dose reduction, and one had two interruptions without any dose change (Fig. 1).

**Fig. 2:**
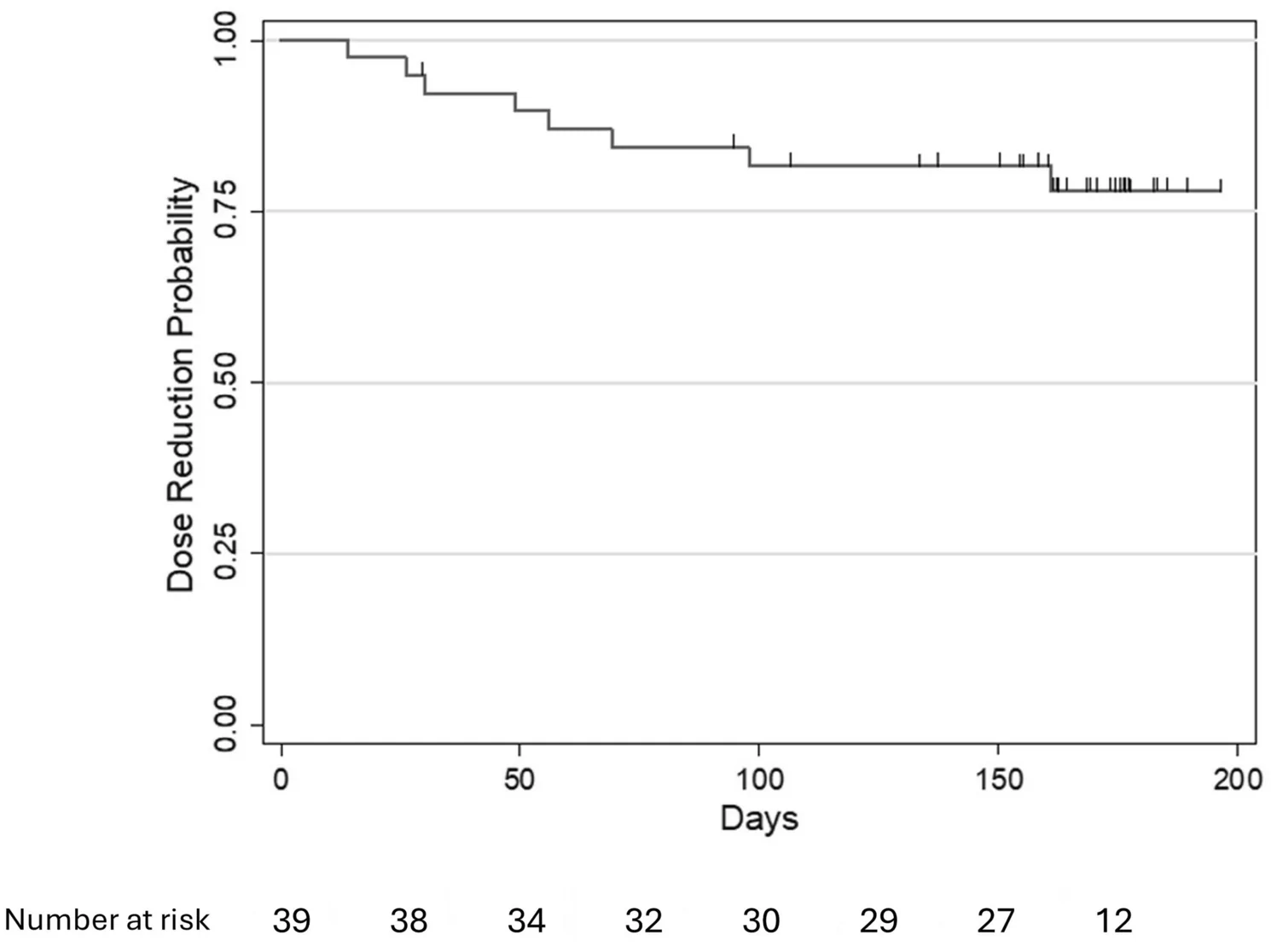
Kaplan–Meier curve of time to first dose reduction due to diarrhea

In four cases, diarrhea was the sole reason for the dose reduction; in another four, it was combined with other unspecified causes. The median time to dose reduction was 52.5 days (IQR 28.0–83.5). Reductions were associated to grade 1 (*n *= 6) or grade 2 (*n* = 3) episodes (Fig. 1). None of the three patients with grade 3 diarrhea had a dose modification. Figure 2 shows the Kaplan–Meier curve for time to dose reduction due to diarrhea.

Four patients (10.3%) temporarily interrupted abemaciclib due to diarrhea (Fig. 1), one of whom had two interruptions. Interruptions were associated with grade 1 (*n *= 2) or grade 2 (*n* = 3) episodes (Fig. 1). The median time to first interruption was 34.5 days (IQR 27.0–44.0), with a median duration of 11.0 days (IQR 5.0–15.0).

#### Patients’ self-care and diarrhea management

Loperamide prescription was the most common strategy for managing diarrhea, reported by over 75% of patients between weeks 2 and 12, with use declining by week 24. Other frequent strategies included increasing fluid intake, astringent diet, and food restrictions—similarly recommended by both the HCPs and the PSP (Supplementary Table 3). Over 60% of the patients found each of these strategies helpful with loperamide being rated as helpful by 100% of patients at week 12.

Patients reported feeling confident and supported in managing diarrhea and their disease. By week 24, over 70% agreed or strongly agreed that the PSP helped them manage diarrhea, adhere to treatment and feel heard and supported (Supplementary Fig. 3).

#### Adherence to treatment (SMAQ)

Over 80% of patients were classified as adherent, with adherence highest at week 2 and lowest at week 24 (Table 3).

**Table 3:** Patients’ adherence (SMAQ) to treatment during follow-up

Time (weeks)	2 weeks	4 weeks	8 weeks	12 weeks	24 weeks
Adherent, n (%)	32 (91.4)	29 (87.9)	28 (84.8)	29 (90.6)	24 (80.0)
Non-adherent, n (%)	3 (8.6)	4 (12.1)	5 (15.1)	3 (9.4)	6 (20.0)
Total *	35 (100)	33 (100)	33 (100)	32 (100)	30 (100)
MD	4	6	5	6	7

* The reported n refers to patients continuing in the study. One patient discontinued at week 4, one at week 12, and four at week 24; therefore, n is adjusted accordingly for follow-up visits; *MD* missing data

#### HRQoL assessment

**Fig. 3:**
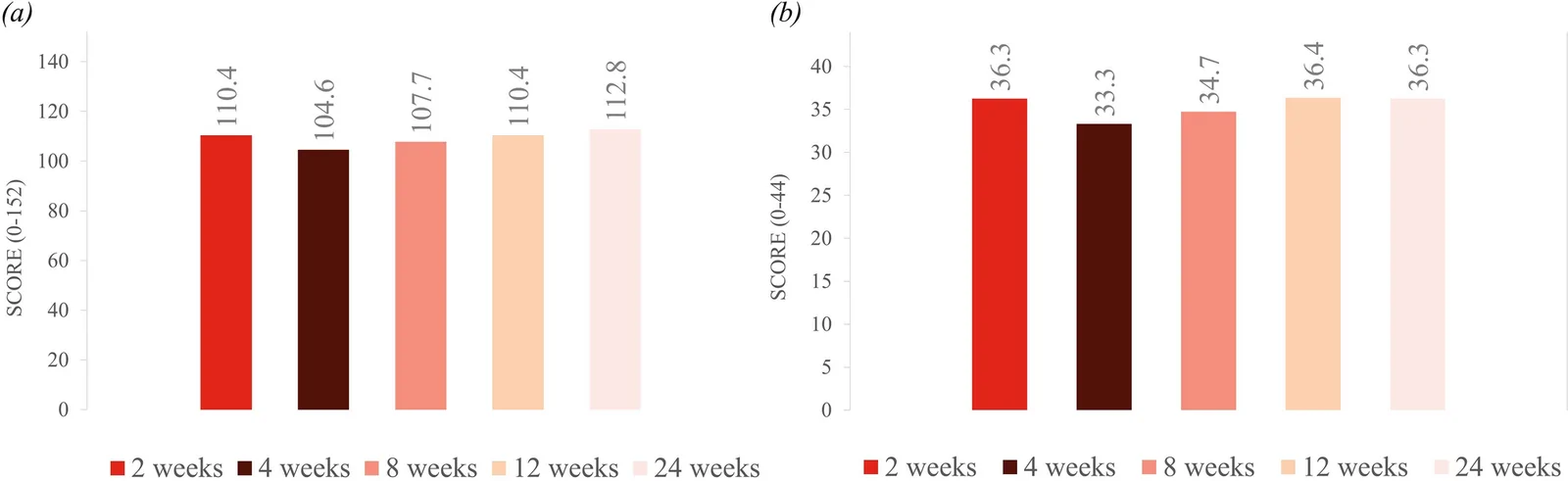
Quality of life of patients (FACIT scores). Panel A shows the total FACIT-D score, and panel B shows the FACIT-D Diarrhea Subscale during follow-up

Mean FACIT-D scores ranged from a low of 104.6 (IQR: 78.0–125.0) at week 4 to a high of 112.8 (IQR: 98.0–132.5) at week 24, on a scale from 0 to 152. Scores declined at weeks 4 and 8 and returned to baseline levels by weeks 12 and 24 (Fig. 3A). Diarrhea subscale scores followed a similar trend, peaking at week 2 (36.4, IQR: 34–42), declining to their lowest at week 8 (33.3, IQR: 25–41) and recovering by the end of follow-up (Fig. 3B).

#### Evaluation of patient persistence, discussions, and satisfaction with the PSP

All patients who continued abemaciclib remained enrolled in the PSP. Two patients discontinued both abemaciclib and the PSP for personal reasons, not reported associated with diarrhea, while two others left the PSP after stopping abemaciclib for reasons other than diarrhea, including one death.

Almost all patients (94.9%) contacted the PSP at least once, discussing diarrhea and nutritional issues in similar proportions (Supplementary Fig. 4).

By week 24, over 70% of the patients reported being satisfied or completely satisfied with the PSP across all evaluated aspects, including information on disease, side effects, diarrhea management, and emotional support (Supplementary Fig. 5 and Supplementary Table 4).

## Discussion

This is the first study to assess abemaciclib and the impact of a PSP within routine clinical practice in Spain. It characterizes patients with locally advanced or MBC receiving abemaciclib and describes the management of diarrhea in this context. Our findings highlight the need for proactive management of diarrhea during the initial weeks of abemaciclib treatment, when most episodes occurred. The PSP may play a key role in supporting adherence and minimizing the clinical impact of this common side effect.

The patient profile was consistent with those described in clinical trials [9, 10] and real-world evidence (RWE) studies [16–19] (age, concomitant treatments, and carcinoma type), suggesting relevance to routine practice in similar settings.

Diarrhea was common (89.7%) and the rates observed were comparable to those reported in clinical trials (98.6% in MONARCH 2 and 82.3% in MONARCH 3) [9, 10]. Similar findings have also been described in real-world evidence; for example, Jacobs et al. conducted a retrospective study in Italy including 39 patients with metastatic breast cancer treated in routine clinical practice where guideline-based diarrhea management strategies were applied and the incidence of diarrhea was 92.3% [20]. Despite these similarities, the occurrence of Grade 3 diarrhea was notably lower in our cohort (7.7% vs 13.4% in MONARCH 2, 9.5% in MONARCH 3, and 16.7% in Jacobs et al.) [9, 10, 20]. Consistent with clinical trials, no grade 4–5 events were observed.

Most participants received the standard dose of abemaciclib, with dose reductions implemented in cases of toxicity, consistent with evidence that dose reductions do not compromise efficacy [11, 21, 22]. Only nine patients (23.1%) required treatment modifications and the rate of dose reductions aligned with previous studies [9, 10, 20, 23]. Notably, no patients discontinued abemaciclib due to diarrhea during follow-up. This result contrasts with clinical trials, where discontinuations due to diarrhea ranged from 1.8 to 2.9% and with Jacobs et al., who reported 10.2% of diarrhea-related discontinuations [9, 10, 20]. Altogether, our results suggest that the PSP may have facilitated effective symptom control and treatment continuation. These results may be particularly relevant given the high rates of diarrhea observed among patients treated with abemaciclib and the known impact of this adverse event on treatment adherence.

The median time to diarrhea onset was 10 days, slightly longer than the 6–8 days reported in clinical trials [9, 10], highlighting the importance of early preventive measures, such as patient education, counseling, and PSPs.

Understanding how patients respond to diarrhea episodes is essential for optimizing symptom control in real-world settings. A 2017 scoping review on the management of toxicities highlighted a lack of studies assessing the effectiveness of self-care actions [24]. In our study, loperamide was the most commonly used strategy, aligning with routine practice [25] and ESMO/SEOM recommendations [26, 27]. Jacobs et al. reported loperamide use in 72% of the participants, matching our findings [20]. Additionally, patients in our cohort commonly adopted dietary modifications to mitigate diarrhea, consistent with previous reports showing that 84% of MBC patients found this approach “successful” [25].

Adherence to oral anticancer treatment is a well-documented challenge that can compromise effectiveness and QoL, often due to side effects [28–31]. In real-world studies of CDK4/6 inhibitors, adherence rates are typically above 80% [31]. In our cohort, adherence remained high (80–91% across visits). While the single-arm design prevents direct attribution to the PSP, maintaining adherence rates similar to those seen in real-world studies is noteworthy, especially in the context of frequent diarrhea.

HRQoL declined slightly in early visits and returned to baseline levels by week 24, mirroring findings from clinical trials [32, 33]. Diarrhea-specific subscale scores showed a similar pattern, reinforcing the benefit of sustained support. Patients also reported high satisfaction with the PSP. Most found it helpful for diarrhea management, adherence, and in emotional support—areas often overlooked in clinical care [34, 35]. The consistent use of the PSP throughout the study and the high consultation rates underscore its acceptability and perceived value. These findings echo in previous literature on the role of PSPs in addressing unmet needs and reducing psychological burden [36–38].

In cancer care, PSPs help capture patients’ concerns [34] and support effective disease management [36, 39] and their potential could be further enhanced when combined with digital health technologies that enable timely monitoring and early intervention for psychological distress [39]. However, such programs remain underused in oncology [12, 24].

This study has several limitations. The observational, single-arm design, and lack of a control group limit the interpretation of the findings and preclude causal inference. Selection bias may exist, as PSP participation could reflect higher motivation or stronger physician–patient relationships, potentially influencing outcomes. The small sample size, although sufficient for descriptive purposes limits the extrapolation of conclusions and external validity. Additionally, not all eligible patients are offered the PSP, and among those who are, not all accept participation. This may introduce selection bias and further limit the generalizability of the findings.

The use of PROs provides valuable insights but is subject to bias and potential missing data. The Hawthorne effect—whereby patients may alter their behavior or reporting due to awareness of being monitored—could have influenced patient behavior and reporting. Moreover, the absence of baseline QoL assessments limits interpretation of changes over time. While the study included several validated questionnaires (SMAQ, FACIT-D), patient satisfaction and diarrhea management were assessed using ad hoc questionnaires that have not undergone formal validation, which may limit the comparability and generalizability of these specific findings.

## Conclusions

The present study highlights the clinical value of PSPs in the context of abemaciclib treatment. Episodes of diarrhea were mostly mild (grade 1–2), and no patients had to discontinue treatment due to this adverse event. Patients reported high satisfaction with the PSP, good adherence, and favorable quality of life, supporting the integration of the PSP in clinical practice. Therefore, programs offering nutritional and adverse event management recommendations can be valuable in improving patient care and preventing treatment discontinuation. These findings may inform the broader implementation of PSPs across clinical settings and contribute to future oncology care strategies.

## Supplementary Information

Below is the link to the electronic supplementary material.

Supplementary file1 (DOCX 439 kb)

## Data Availability

The data that support the findings of this study are not publicly available due to ethical and legal restrictions related to patient confidentiality.
