# Supplementary material for: Clinical experience and satisfaction in patients with advanced breast cancer participating in the abemaciclib patient support program in Spain: a prospective observational study
Source: Clin Transl Oncol. 2026 Mar 12;28(9):3862–71. doi: 10.1007/s12094-026-04267-y (PMC13499750; doi:10.1007/s12094-026-04267-y)
Supplement: Supplementary file 1 [file 12094_2026_4267_MOESM1_ESM.docx]

**Supplementary material**

Supplementary Fig. 1 Study design

**Supplementary Table 1:** Self-care practices and self-management of diarrhea questionnaire

**SELF-CARE PRACTICES AND SELF-MANAGEMENT OF DIARRHOEA**

This questionnaire is composed by two sections where we will ask you about:

- Section A: In case you have had diarrhoea, how you have managed it.
- Section B: How do you feel about the treatment and management of diarrhoea.

It is very important that you answer all the questions. **Mark your answers with an X**. Make sure that the copy sheet (yellow sheet) is well marked.

| A. **A) SELF-CARE AND SELF-MANAGEMENT DURING DIARRHOEAL EPISODES:**   Answer the following table **if you have had diarrhoea**. Otherwise, skip to section B | | | | | | | | |  |
| --- | --- | --- | --- | --- | --- | --- | --- | --- | --- |
| If you presented diarrhoea from the last visit, **how you managed it?** *Please, mark with an X the measures adopted* | | | I received this advice from: | | | | Did it help your diarrhoea? | | |
|  |  |  | Physician, nurse or pharmacist | | PSP | None |  |  |  |
| ☐ | | Increase fluid intake | ☐ | | ☐ | ☐ | ☐ Yes ☐ No | |  |
| ☐ | | Change of diet (astringent diet) | ☐ | | ☐ | ☐ | ☐ Yes ☐ No | |  |
| ☐ | | Diet restrictions | ☐ | | ☐ | ☐ | ☐ Yes ☐ No | |  |
| ☐ | | Use of prescribed loperamide | ☐ | | ☐ | ☐ | ☐ Yes ☐ No | |  |
| ☐ | | Use of other prescribed drugs. Specify:___________ | ☐ | | ☐ | ☐ | ☐ Yes ☐ No | |  |
|  |  | Use of other prescribed drugs. Specify:__________ | ☐ | | ☐ | ☐ | ☐ Yes ☐ No | |  |
|  |  | Use of other prescribed drugs. Specify:___________ | ☐ | | ☐ | ☐ | ☐ Yes ☐ No | |  |
| ☐ | | Use of loperamide without medical prescription (OTC) | ☐ | | ☐ | ☐ | ☐ Yes ☐ No | |  |
| ☐ | | Use of dietary supplements | ☐ | | ☐ | ☐ | ☐ Yes ☐ No | |  |
| ☐ | | Use of herbal remedies | ☐ | | ☐ | ☐ | ☐ Yes ☐ No | |  |
| ☐ | | Rest and reduce normal activity | ☐ | | ☐ | ☐ | ☐ Yes ☐ No | |  |
|  | B. **B) CURRENTLY, YOU FEEL…**   *Please, mark with an X your answer to each one of the questions.* | | | | | | |  |  |
|  | I am confident to manage diarrhoea episodes when they occur | | | ☐ Strongly disagree  ☐ Disagree  ☐ Neither agree nor disagree.  ☐ Agree  ☐ Strongly agree  ☐ Not applicable | | | |  |  |
|  | I am confident to contact with physician/nurse responsible for my cancer treatment if I present diarrhoea episodes | | | ☐ Strongly disagree  ☐ Disagree  ☐ Neither agree nor disagree.  ☐ Agree  ☐ Strongly agree  ☐ Not applicable | | | |  |  |
|  | I am confident to contact with PSP if I present diarrhoea episodes | | | ☐ Strongly disagree  ☐ Disagree  ☐ Neither agree nor disagree.  ☐ Agree  ☐ Strongly agree  ☐ Not applicable | | | |  |  |
|  | I believe that, apart from medicines, there are other important measures (e.g. diet and lifestyle) in the management of diarrhoea. | | | ☐ Strongly disagree  ☐ Disagree  ☐ Neither agree nor disagree.  ☐ Agree  ☐ Strongly agree  ☐ Not applicable | | | |  |  |
|  | I can recognise the intensity of diarrhoea episodes when I have it and I know when I should call for help | | | ☐ Strongly disagree  ☐ Disagree  ☐ Neither agree nor disagree.  ☐ Agree  ☐ Strongly agree  ☐ Not applicable | | | |  |  |
|  | The PSP has helped me to manage diarrhoea episodes | | | ☐ Strongly disagree  ☐ Disagree  ☐ Neither agree nor disagree.  ☐ Agree  ☐ Strongly agree  ☐ Not applicable | | | |  |  |
|  | Participating in the PSP helps me to take my medicine as I have been instructed. | | | ☐ Strongly disagree  ☐ Disagree  ☐ Neither agree nor disagree.  ☐ Agree  ☐ Strongly agree  ☐ Not applicable | | | |  |  |
|  | Participating in the PSP makes me feel listened and supported regarding my disease | | | ☐ Strongly disagree  ☐ Disagree  ☐ Neither agree nor disagree.  ☐ Agree  ☐ Strongly agree  ☐ Not applicable | | | |  |  |

**Supplementary Table 2** Patient satisfaction with PSP questionnaire

**PATIENT SATISFACTION WITH PSP**

**How do you feel about…?** *Please, mark with an X the answer you consider appropriate.*

**Participation on the PSP:**

☐ Totally unsatisfied ☐ Unsatisfied ☐ Indifferent ☐ Satisfied ☐ Totally satisfied

**Information about the disease given by PSP:**

☐ Totally unsatisfied ☐ Unsatisfied ☐ Indifferent ☐ Satisfied ☐ Totally satisfied

**Information about adverse effects I may experiment during treatment**

☐ Totally unsatisfied ☐ Unsatisfied ☐ Indifferent ☐ Satisfied ☐ Totally satisfied

**Information about how to manage diarrhoea episodes while taking abemaciclib:**

☐ Totally unsatisfied ☐ Unsatisfied ☐ Indifferent ☐ Satisfied ☐ Totally satisfied

**Information about the severity of diarrhoea episodes:**

☐ Totally unsatisfied ☐ Unsatisfied ☐ Indifferent ☐ Satisfied ☐ Totally satisfied

**Information about dietary habits to help managing diarrhoea episodes:**

☐ Totally unsatisfied ☐ Unsatisfied ☐ Indifferent ☐ Satisfied ☐ Totally satisfied

**Information about where and when to call for help/health services while having diarrhoea:**

☐ Totally unsatisfied ☐ Unsatisfied ☐ Indifferent ☐ Satisfied ☐ Totally satisfied

**Emotional support provided by PSP professionals:**

☐ Totally unsatisfied ☐ Unsatisfied ☐ Indifferent ☐ Satisfied ☐ Totally satisfied

**Supplementary Figure 2** Treatment modifications due to diarrhea (swimmer plot)

Supplementary Table 3 Patient´s self-care and diarrhea management ad-hoc questionnaire

| **Time in weeks (patients)** | **2 weeks**  (n = 21) | | **4 weeks**  (n = 25) | | **8 weeks**  (n = 23) | | **12 weeks**  (n = 20) | | **24 weeks**  (n = 17) | |
| --- | --- | --- | --- | --- | --- | --- | --- | --- | --- | --- |
| **3** | **n** | **%** | **n** | **%** | **n** | **%** | **n** | **%** | **n** | **%** |
| **Increase fluid intake** | 14 | 66.67 | 17 | 68.00 | 16 | 69.57 | 13 | 65.00 | 12 | 70.59 |
| *Recommended by the HCP* | 10 | 47.62 | 11 | 44.00 | 13 | 56.52 | 11 | 55.00 | 8 | 47.06 |
| *Recommended by the PSP* | 9 | 42.86 | 11 | 44.00 | 12 | 52.17 | 7 | 35.00 | 7 | 41.18 |
| *Recommended by other sources* | 4 | 19.05 | 2 | 8.00 | 0 | 0.00 | 2 | 10.00 | 2 | 11.76 |
| *Recommendation was helped* | 11 | 78.57 | 16 | 94.12 | 12 | 75.00 | 11 | 84.62 | 7 | 58.33 |
| **Diet change (to an astringent diet)** | 12 | 57.14 | 17 | 68.00 | 15 | 65.22 | 15 | 75.00 | 11 | 64.71 |
| *Recommended by the HCP* | 7 | 33.33 | 9 | 36.00 | 9 | 39.13 | 12 | 60.00 | 4 | 23.53 |
| *Recommended by the PSP* | 8 | 38.10 | 12 | 48.00 | 9 | 39.13 | 9 | 45.00 | 6 | 35.29 |
| *Recommended by other sources* | 2 | 9.52 | 4 | 16.00 | 1 | 4.35 | 2 | 10.00 | 3 | 17.65 |
| *Recommendation was helped* | 9 | 75.00 | 15 | 88.24 | 10 | 66.67 | 13 | 86.67 | 5 | 45.45 |
| **Diet restrictions** | 12 | 57.14 | 13 | 52.00 | 8 | 34.78 | 12 | 60.00 | 7 | 41.18 |
| *Recommended by the HCP* | 6 | 28.57 | 6 | 24.00 | 5 | 21.74 | 8 | 40.00 | 1 | 5.88 |
| *Recommended by the PSP* | 6 | 28.57 | 10 | 40.00 | 5 | 21.74 | 5 | 25.00 | 3 | 17.65 |
| *Recommended by other sources* | 2 | 9.52 | 3 | 12.00 | 1 | 4.35 | 4 | 20.00 | 2 | 11.76 |
| *Recommendation was helped* | 10 | 83.33 | 11 | 84.62 | 5 | 62.50 | 11 | 91.67 | 4 | 57.14 |
| **Use of prescribed loperamide** | 16 | 76.19 | 19 | 76.00 | 19 | 82.61 | 15 | 75.00 | 10 | 58.82 |
| *Recommended by the HCP* | 13 | 61.90 | 17 | 68.00 | 17 | 73.91 | 14 | 70.00 | 7 | 41.18 |
| *Recommended by the PSP* | 11 | 52.38 | 11 | 44.00 | 9 | 39.13 | 4 | 20.00 | 2 | 11.76 |
| *Recommended by other sources* | 0 | 0.00 | 2 | 8.00 | 0 | 0.00 | 0 | 0.00 | 1 | 5.88 |
| *Recommendation was helped* | 15 | 93.75 | 18 | 94.74 | 16 | 84.21 | 15 | 100.00 | 7 | 70.00 |
| **Use of another prescribed medication** | 1 | 4.76 | 1 | 4.00 | 0 | 0.00 | 1 | 5.00 | 1 | 5.88 |
| *Recommended by the HCP* | 1 | 4.76 | 1 | 4.00 | - | - | 0 | 0.00 | 1 | 5.88 |
| *Recommended by the PSP* | 0 | 0.00 | 0 | 0.00 | - | - | 0 | 0.00 | 0 | 0.00 |
| *Recommended by other sources* | 0 | 0.00 | 0 | 0.00 | - | - | 0 | 0.00 | 0 | 0.00 |
| *Recommendation was helped* | 1 | 50.00 | 1 | 50.00 | - | - | 0 | 0.00 | 1 | 100.00 |
| **Use of non-prescribed loperamide** | 4 | 19.05 | 4 | 16.00 | 4 | 17.39 | 5 | 25.00 | 4 | 23.53 |
| *Recommended by the HCP* | 1 | 4.76 | 0 | 0.00 | 1 | 4.35 | 0 | 0.00 | 2 | 11.76 |
| *Recommended by the PSP* | 0 | 0.00 | 0 | 0.00 | 1 | 4.35 | 0 | 0.00 | 1 | 5.88 |
| *Recommended by other sources* | 2 | 9.52 | 3 | 12.00 | 3 | 13.04 | 3 | 15.00 | 1 | 5.88 |
| *Recommendation was helped* | 3 | 75.00 | 1 | 25.00 | 0 | 0.00 | 3 | 60.00 | 2 | 50.00 |
| **Use of dietary supplements** | 5 | 23.81 | 7 | 28.00 | 3 | 13.04 | 4 | 20.00 | 3 | 17.65 |
| *Recommended by the HCP* | 2 | 9.52 | 1 | 4.00 | 0 | 0.00 | 0 | 0.00 | 0 | 0.00 |
| *Recommended by the PSP* | 1 | 4.76 | 1 | 4.00 | 0 | 0.00 | 0 | 0.00 | 0 | 0.00 |
| *Recommended by other sources* | 3 | 14.29 | 5 | 20.00 | 3 | 13.04 | 2 | 10.00 | 2 | 11.76 |
| *Recommendation was helped* | 3 | 60.00 | 2 | 28.57 | 1 | 33.33 | 1 | 25.00 | 1 | 33.33 |
| **Use of medicinal herbs/plants** | 6 | 28.57 | 5 | 20.00 | 3 | 13.04 | 6 | 30.00 | 3 | 17.65 |
| *Recommended by the HCP* | 2 | 9.52 | 1 | 4.00 | 0 | 0.00 | 2 | 10.00 | 0 | 0.00 |
| *Recommended by the PSP* | 1 | 4.76 | 2 | 8.00 | 0 | 0.00 | 0 | 0.00 | 0 | 0.00 |
| *Recommended by other sources* | 2 | 9.52 | 3 | 12.00 | 3 | 13.04 | 2 | 10.00 | 1 | 5.88 |
| *Recommendation was helped* | 4 | 66.67 | 2 | 40.00 | 1 | 33.33 | 2 | 33.33 | 1 | 33.33 |
| **Rest and reduction of daily activities** | 6 | 28.57 | 10 | 40.00 | 10 | 43.48 | 6 | 30.00 | 4 | 23.53 |
| *Recommended by the HCP* | 3 | 14.29 | 7 | 28.00 | 3 | 13.04 | 2 | 10.00 | 0 | 0.00 |
| *Recommended by the PSP* | 3 | 14.29 | 6 | 24.00 | 4 | 17.39 | 1 | 5.00 | 0 | 0.00 |
| *Recommended by other sources* | 3 | 14.29 | 3 | 12.00 | 5 | 21.74 | 3 | 15.00 | 1 | 5.88 |
| *Recommendation was helped* | 4 | 66.67 | 8 | 80.00 | 7 | 70.00 | 3 | 50.00 | 2 | 50.00 |

*HCP* health care provider, *PSP* patient support program

Supplementary Fig. 3 How patient feels related to treatment and diarrhea management

**Supplementary Fig. 4** Number of calls (a) and topics discussed (B) during follow-up times

A

B

Supplementary Fig. 5 Patient´s satisfaction level regarding several PSP-related statements at week 24

| **Supplementary Table 4** Patients’ satisfaction with PSP during study treatment: How do you feel about…? | | | | | | |
| --- | --- | --- | --- | --- | --- | --- |
|  |  | Completely disagree | Disagree | not agree/not disagree | Agree | Completely Agree |
| Participation on the PSP | 2 weeks | 0.0 | 5.6 | 8.3 | 47.2 | 38.9 |
|  | 4 weeks | 5.6 | 0.0 | 13.9 | 55.6 | 25.0 |
|  | 8 weeks | 0.0 | 5.6 | 16.7 | 50.0 | 27.8 |
|  | 12 weeks | 2.7 | 5.4 | 8.1 | 62.2 | 21.6 |
|  | 24 weeks | 2.9 | 5.7 | 14.3 | 48.6 | 28.6 |
| Information about the disease given by PSP | 2 weeks | 0.0 | 8.3 | 5.6 | 47.2 | 38.9 |
|  | 4 weeks | 0.0 | 0.0 | 8.3 | 66.7 | 25.0 |
|  | 8 weeks | 0.0 | 5.6 | 5.6 | 61.1 | 27.8 |
|  | 12 weeks | 2.7 | 5.4 | 2.7 | 64.9 | 24.3 |
|  | 24 weeks | 2.8 | 2.8 | 13.9 | 50.0 | 30.6 |
| Information about adverse effects I may experiment during treatment | 2 weeks | 0.0 | 5.6 | 8.3 | 38.9 | 47.2 |
|  | 4 weeks | 0.0 | 5.6 | 8.3 | 66.7 | 19.4 |
|  | 8 weeks | 2.8 | 5.6 | 8.3 | 55.6 | 27.8 |
|  | 12 weeks | 2.7 | 5.4 | 5.4 | 59.5 | 27.0 |
|  | 24 weeks | 5.7 | 0.0 | 5.7 | 51.4 | 37.1 |
| Information about how to manage diarrhoea episodes while taking abemaciclib | 2 weeks | 0.0 | 5.6 | 5.6 | 55.6 | 33.3 |
|  | 4 weeks | 0.0 | 2.8 | 5.6 | 66.7 | 25.0 |
|  | 8 weeks | 0.0 | 0.0 | 16.7 | 50.0 | 33.3 |
|  | 12 weeks | 0.0 | 5.4 | 10.8 | 59.5 | 24.3 |
|  | 24 weeks | 5.7 | 0.0 | 11.4 | 51.4 | 31.4 |
| Information about the severity of diarrhoea episodes | 2 weeks | 2.8 | 5.6 | 11.1 | 50.0 | 30.6 |
|  | 4 weeks | 0.0 | 5.6 | 11.1 | 63.9 | 19.4 |
|  | 8 weeks | 2.9 | 0.0 | 14.3 | 60.0 | 22.9 |
|  | 12 weeks | 0.0 | 5.4 | 10.8 | 54.1 | 29.7 |
|  | 24 weeks | 5.7 | 0.0 | 17.1 | 42.9 | 34.3 |
| Information about dietary habits to help managing diarrhoea episodes | 2 weeks | 5.6 | 5.6 | 11.1 | 47.2 | 30.6 |
|  | 4 weeks | 0.0 | 2.8 | 11.1 | 61.1 | 25.0 |
|  | 8 weeks | 2.8 | 0.0 | 16.7 | 58.3 | 22.2 |
|  | 12 weeks | 0.0 | 5.4 | 5.4 | 67.6 | 21.6 |
|  | 24 weeks | 2.9 | 2.9 | 14.3 | 48.6 | 31.4 |
| Information about where and when to call for help/health services while having diarrhoea | 2 weeks | 2.8 | 2.8 | 11.1 | 50.0 | 33.3 |
|  | 4 weeks | 0.0 | 8.3 | 2.8 | 66.7 | 22.2 |
|  | 8 weeks | 0.0 | 0.0 | 13.9 | 63.9 | 22.2 |
|  | 12 weeks | 0.0 | 5.4 | 8.1 | 67.6 | 18.9 |
|  | 24 weeks | 2.9 | 2.9 | 14.3 | 51.4 | 28.6 |
| Emotional support provided by PSP professionals | 2 weeks | 2.8 | 5.6 | 19.4 | 44.4 | 27.8 |
|  | 4 weeks | 0.0 | 5.6 | 13.9 | 61.1 | 19.4 |
|  | 8 weeks | 0.0 | 2.8 | 16.7 | 55.6 | 25.0 |
|  | 12 weeks | 0.0 | 7.9 | 13.2 | 55.3 | 23.7 |
|  | 24 weeks | 2.9 | 5.7 | 20.0 | 40.0 | 31.4 |
